# Amyloid β and cardiovascular risk factors promote endothelial cell apoptotic and mitochondrial stress, blood‐brain barrier dysfunction and angiogenesis impairment

**DOI:** 10.1002/alz.087125

**Published:** 2025-01-03

**Authors:** Silvia Fossati

**Affiliations:** ^1^ Alzheimer’s Center at Lewis Katz School of Medicine, Temple University, Philadelphia, PA USA

## Abstract

**Background:**

Brain endothelial cell (EC) stress, including that induced by vascular amyloid β (Aβ) deposits in cerebral amyloid angiopathy (CAA) and Alzheimer’s disease (AD), contributes to cerebral blood flow impairment, blood brain barrier (BBB) damage, neurovascular unit dysfunction, microhemorrhages and hypoperfusion, precipitating neurodegeneration and neuroinflammation processes. Epidemiological and experimental evidence suggests that hyperhomocysteinemia (Hhcy) contributes to increasing AD risk as well as CAA pathology. However, the cellular and molecular mechanisms through which Aβ and Hhcy drive EC and BBB dysfunction, whether the molecular effects of these challenges are additive or independent, and possible therapeutic strategies, remain to be determined.

**Methods:**

Our work analyzed specific mitochondria‐ and death receptor‐mediated pathways in human cerebral microvascular ECs challenged with Aβ, high Hcy, or their combination. We also assessed the effects of these challenges, alone or combined, on the loss of BBB Resistance, junction proteins expression and structure and angiogenic activity, as well as the potential protective effects of carbonic anhydrase inhibitors.

**Results:**

Our data demonstrates that Aβ activates death receptors 4/5 (DR4/5)‐ and mitochondria‐mediated apoptotic pathways in human cerebral microvascular ECs and promotes loss of mitochondrial respiratory function (OXPHOS). Hhcy potentiates the Aβ‐mediated activation of both the extrinsic and intrinsic mitochondria‐mediated apoptotic pathways, the loss EC barrier permeability, a deregulation of multiple junction proteins and the impairment in VEGF‐A/VEGF‐receptor signaling and angiogenic capabilities. However, Hhcy does not affect endothelial Aβ‐mediated OXPHOS impairment and metabolic defects. Carbonic anhydrase inhibitors successfully improve EC apoptotic outcomes and microvascular fitness both *in vitro* and *in vivo*.

**Conclusion:**

This data highlights new and specific molecular mechanisms that drive endothelial and BBB pathology in AD/CAA in the presence or absence of a comorbid cerebrovascular condition such as Hhcy. Moreover, we discovered a new potential therapeutic target, carbonic anhydrase, whose inhibition can revert the cerebrovascular effects of Aβ *in vitro* and *in vivo*.

